# Border control: Anatomical origins of the thymus medulla

**DOI:** 10.1002/eji.201545829

**Published:** 2015-08-07

**Authors:** Graham Anderson, William E. Jenkinson

**Affiliations:** ^1^ MRC Centre for Immune RegulationInstitute for Biomedical Research, Medical School, University of BirminghamBirminghamUK

**Keywords:** Central tolerance, Epithelial cells, Stem cells, T cell, Thymus

## Abstract

The thymus is an anatomically compartmentalized primary lymphoid organ that fosters the production of self‐tolerant T cells. The thymic cortex provides a specialized microenvironment in which cortical thymic epithelial cells (cTECs) support the positive selection and further differentiation of self‐MHC‐restricted thymocytes. Following their migration into the medulla, positively selected thymocytes are further screened for self‐reactivity, which involves both negative selection and Foxp3^+^ regulatory T cell generation via interactions with medullary thymic epithelial cells (mTECs). Given the importance of both cortical and medullary microenvironments for T cell development, studies that address the developmental origins of cTECs and mTECs are important in understanding the processes that shape the developing T cell receptor repertoire, and reduce the frequency of self‐reactive T cells that initiate autoimmune disease. In this issue of the *European Journal of Immunology*, Onder et al. [Eur. J. Immunol. 2015. 45: 2218‐2231] identified a subset of podoplanin^+^ mTECs in mice that reside at the corticomedullary junction (CMJ), show that their development is important to establish self‐tolerance, and require the presence of self‐reactive T cells. Collectively, their findings highlight the CMJ as a potential repository for precursors of the mTEC lineage, and provide a better understanding of thymus medulla formation.

Intrathymic αβ T cell development relies upon a fine balance operating between the opposing outcomes of positive and negative selection. The drive to select functionally diverse T cell clones, required for effective T cell‐mediated immunity, must be tempered by the need to restrict the development and activation of randomly generated autoreactive T cells. Critically, anatomically, and functionally distinct epithelial cells of the thymus differentially regulate these opposing developmental outcomes 1. Cortical thymic epithelial cells (cTECs) drive positive selection. In contrast, medullary thymic epithelial cells (mTECs) are the primary orchestrating cell‐type responsible for the enforcement of intrathymic T cell tolerance, driving both the negative selection of autoreactive clones and the maturation of thymic regulatory T cells. The importance of understanding mTEC development is highlighted by the manifestation of autoimmune disease associated with aberrant thymic medullary development and function. A prime example is the human autoimmune disease APECED, in which defective functional development of mature mTECs correlates with mutations in the autoimmune regulator (Aire) 2. Harnessing the potential of thymic epithelial progenitor cells, particularly with regard to the corrective restoration of defective mTECs, presents an attractive avenue for cellular therapeutic manipulation of central tolerance mechanisms. However, the potential of such approaches currently remain hampered by an incomplete knowledge of the basic biology of mTEC generation and maintenance. In this issue of the *European Journal of Immunology*, a study by Onder et al. 3 provides new information extending our current knowledge of mTEC precursor populations in the adult thymus, and shed further light on this important but elusive cell‐ type.

The functional capacity of mTECs to uniquely shape T cell development is driven by several key functional characteristics. For instance, the production of chemokine ligands, including CCL19 and CCL21, has been shown to regulate the accumulation of newly positively selected CCR7^+^ CD4^+^ and CD8^+^ single‐positive thymocytes within medullary regions in the murine thymus 4. In addition, the ectopic expression of peripheral tissue antigens occurring via both Aire‐dependent and –independent mechanisms, underpins the ability of mTECs to enforce the deletion of self‐reactive T cells prior to their release into the peripheral circulation 5. The exquisite influence of such mechanisms on the enforcement of central tolerance is revealed in murine models demonstrating that the loss of single Aire‐dependent antigens in mTECs, such as the eye‐related interphotoreceptor retinoid‐binding protein or the stomach‐related antigen Mucin 6, consequentially leads to the manifestation of organ‐specific autoimmunity 6, 7. Interestingly, the adult mTEC compartment displays a surprisingly high degree of cell turnover, implying that constant replenishment of functionally mature mTECs must occur in order to maintain effective central tolerance throughout life. However, as yet the full identity, including the degree of heterogeneity, and intrathymic localization of mTEC precursor pools remains incompletely understood.

The maturation of mTEC compartments is intrinsically linked to T cell development. Specifically, tumor necrosis factor superfamily (TNFSF) ligand expression by positively selected CD4^+^ thymocytes drives mTEC maturation via signaling through TNFSF receptors, including CD40, lymphotoxin‐β receptor and receptor activator of nuclear factor‐κ B (RANK) 8, 9, 10, 11. Given the dependency of mTEC development upon CD4^+^ thymocyte interactions, the intrathymic localization of mTEC precursors is likely a key determinant involved in the generation and maintenance of functional medullary epithelium. Although previous reports have described the initial formation of large medullary thymic regions arising from the confluence of epithelial islets, each derived from single clonal mTEC precursors 12, the characteristics and positioning of mTEC precursors in the adult steady‐state thymus have yet to be fully elucidated.

In this issue, Onder and colleagues describe the use of several interesting mouse strains to analyze the development, make up, and functional importance of the adult mTEC compartment 3. First, using a Ccl19‐Cre eYFP‐based fate mapping system to label and track the fate of any cells that have expressed Ccl19 at any stage, they show that within total TECs, eYFP expression selectively identifies the mTEC compartment, with cTECs lacking eYFP expression. These data correlate with previous studies demonstrating that CCL19 is detected predominantly within the thymic medulla rather than cortex 13. Next, the authors used an MHC class II‐restricted TCR transgenic system, in which thymocytes recognize the 614–629 amino acid sequence derived from cardiac myosin heavy chain alpha (MYHCA), and measured the impact on the mTEC compartment when the MYHCA614‐629 epitope was expressed by Ccl19‐Cre^+^ TECs. These experiments showed that the intrathymic recognition of self‐antigens — in this case, the engineered MYHCA614‐629 epitope — that occurs during thymic selection enhances thymus medulla development, and increases mTEC numbers. Specifically, Onder et al. show that the expansion of the thymic medulla involved a TEC subset residing at the corticomedullary junction (CMJ), which lacked markers typical of mature mTECs, including CD80 and UEA1. Interestingly, these cells could be identified on the basis of their expression of podoplanin (PDPN), a type 1 integral membrane protein that has previously been identified in the mouse using the monoclonal antibody 8.1.1 14. Moreover, through the use of a *Pdpn‐Cre/R26‐eyfp* fate mapping approach to further analyze the nature of PDPN^+^ TECs, they showed that the majority of EpCAM^+^eYFP^+^ cells were contained within medullary areas, and displayed an mTEC‐like phenotype. From these observations, the authors propose that a TEC subset anatomically positioned at the CMJ, and defined by PDPN expression, represents an mTEC‐committed precursor, termed here “junctional TEC” or “jTEC,” and preferentially gives rise to mature mTECs. Given the importance of nuclear factor κ B (NF‐κB) signaling in the development of the mTEC compartment for tolerance induction, the authors then show that the deletion of nuclear factor κ B inducing kinase (NIK), a key regulator of alternative NF‐κB signaling in Ccl19‐Cre^+^ TECs, results in a blockade in mTEC development (Fig. 1). Transcriptomic microarray analysis of PDPN^+^ jTECs, NIK‐deficient TECs, and PDPN^−^ mTECs revealed a strikingly close relationship of PDPN^+^ jTECs to NIK‐deficient TECs, whereas PDPN^−^ mTECs displayed a distinct molecular profile. Interestingly several NF‐κB signaling related genes, including *Ccl21*, *Cxcl12*, and *IL‐7* were expressed at relatively higher levels in PDPN^+^ jTECs compared to PDPN^−^ mTECs, whereas expression of *Aire*, *Aire‐*dependent peripheral tissue antigens, and some NF‐κB signaling related genes such as *Cd40* and *Tnfrsf11a* were upregulated in PDPN^−^ mTEC compared to PDPN^+^ jTECs. Consistent with the role of intact medullary compartments for the enforcement of central tolerance, the loss of mature mTEC following Ccl19‐Cre^+^‐driven deletion of NIK in TECs, predisposed mice to T‐cell‐mediated autoimmunity.

**Figure 1:**
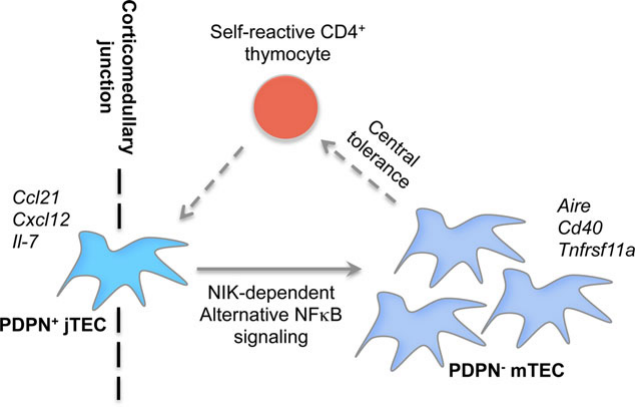
Model of the contribution of PDPN^+^ junctional thymic epithelial cell (jTEC) precursors to the formation of mTEC compartments based on the study by Onder et al. 3. PDPN^+^ jTECs, located at the corticomedullary junction, undergo regulation by self‐reactive CD4^+^8^−^ thymocytes and give rise to NIK‐dependent PDPN^−^ mTECs that are critical for the enforcement of central tolerance via reciprocal crosstalk with self‐reactive CD4^+^8^−^ T cells. Solid arrow, developmental progression of jTEC; dashed arrow, mTEC‐thymocyte crosstalk.

The findings of Onder et al. 3 add another piece to the puzzle regarding our current understanding of events that control thymus medulla development and maintenance. Following the initial description of mTEC‐restricted precursors capable of forming individual medullary clonal islets 12, bipotent precursors that give rise to both cTEC and mTEC lineages expressing the TEC‐defining transcription factor Foxn1, have been identified in the fetal and postnatal thymus in mice 15, 16, 17. A recent study has further reported the presence of rare adult thymic stromal cells, which under specific in vitro culture conditions, form spheroid structures proposed to contain bipotent TEC precursors 18. Interestingly, such sphere‐forming cells lack epithelial markers such as CD326 and possess the in vitro capacity to generate both cTEC‐ and mTEC‐committed cells in a Foxn1‐independent fashion 18. Additional studies in mouse embryos have shown that the mTEC lineage arises from an intermediate precursor population displaying hallmarks characteristic of cTEC lineage cells, including CD205, the thymoproteasomal subunit β5t, and high levels of IL‐7 19, 20, 21. While it is unclear whether a similar process occurs beyond embryonic stages, the mTEC compartment in the adult thymus is highly dynamic. For example, estimates of mTEC turnover time are in the range of 1–2 weeks 22, which is suggestive of the need for continual replacement of mTECs via one or more precursor population(s). Perhaps most significantly in relation to the continued production of mTECs in adult life, studies by Sekai et al. 23 revealed the presence of mTEC‐restricted stem cells that are defined by coexpression of high levels of Claudin‐3 and ‐4, and SSEA‐1, a marker associated with stemness in a variety of tissues. Critically, such SSEA‐1^+^ mTEC stem cells possess the capacity to generate mature mTECs throughout life and thereby ensure the maintenance of central tolerance. Importantly however, the relationship of these mTEC stem cells to the PDPN^+^ jTEC population in adult mice as described by Onder et al. 3 remains unclear. Indeed, whether jTECs represent the same mTEC stem cell pool, or a downstream relation, remains to be ascertained. Indeed, while the experiments presented by Onder et al. imply that PDPN^+^ jTECs possess mTEC precursor potential, further investigation of the precursor–product relationship of jTECs and mature mTECs, including at a clonal level, and the degree to which they contribute to all mTEC subsets in the steady‐state thymus will extend our knowledge of this newly described mTEC subpopulation. Future experiments aimed at determining the self‐renewal potential and turnover of jTECs will provide interesting avenues of further research.

Recent advances in our understanding of mTEC heterogeneity have emerged over recent years, and it will be important to relate to the identification of mTEC precursors that lie downstream of mTEC stem cells. For example, mTECs can be subdivided into mTEC^lo^ and mTEC^hi^ subsets on the basis of MHC class II and CD80 expression, and while a precursor–product relationship has been identified in these cells 24, the frequency of mTEC progenitors within the mTEC^lo^ subset has not been determined. Given the importance of RANK signaling in the development of the mTEC precursors that give rise to Aire^+^ mTECs 24, a clearer definition of the frequency, phenotypic, and developmental properties of RANK^+^ mTEC precursors in relation to jTECs and mTECs, will be important. In addition, the mTEC^lo^ compartment has been shown to include CCL21^+^ mTECs, which may represent mature cells without the capacity to generate mTEC^hi^ cells 25, while a terminal differentiation stage in mTEC development has been suggested, in which Aire^+^ mTEC^hi^ cells downregulate MHC class II and CD80, essentially reacquiring the mTEC^lo^ phenotype 26. These data may be particularly interesting given the finding by Onder et al. that jTEC express Ccl21 at relatively high levels and MHC class II at relatively low levels, compared to mature mTEC 3. Further studies are required to determine whether jTECs, or other additional mTEC precursor populations, are capable of efficiently generating all mTEC lineages or whether specific precursor pools preferentially form defined mTEC subcompartments.

Interestingly, while the activity of SSEA‐1^+^ mTEC stem cells is reported to decline in a post‐positive selection T cell‐dependent manner post birth 23, the activity and expansion of jTECs would appear to be positively regulated by interactions with self‐reactive CD4^+^ single‐positive thymocytes 3. These opposing findings raise the interesting possibility that the activity of rare lifelong mTEC stem cells is reduced once a sufficient medullary size is reached, as dictated by T‐cell feedback, in order to help maintain the stem cell compartment, and the maintenance of the mTEC compartment falls predominantly under the control of downstream mTEC precursors operating in a manner akin to transit amplifying cells 27. In this regard, it could be predicted that the activity of heterogeneous mTEC precursors may be differentially sensitive to alterations in T‐cell development. In this regard, analysis of mTEC precursor compartments in situations of acute thymic atrophy and recovery, for instance postimmunoablative therapy, warrants further investigation.

The potential positioning of mTEC precursor populations at the CMJ fits well with earlier reports proposing that the CMJ acts as a potential TEC stem cell site 28. Indeed, Onder et al. speculate that the anatomical localization of mTEC precursors at the CMJ ensures that such cells are ideally placed to receive maturation signals from CD4^+^ thymocytes crossing from the cortex into the medulla 3. Interestingly, this raises the question of whether the CMJ as an anatomical structure is in itself an important site for the maintenance of mTEC precursors, perhaps due to maintenance of precursor cell polarity and asymmetric cell division, or alternatively whether the CMJ is simply a product of the positioning of mTEC precursor cells themselves, and the subsequent development and expansion of their progeny. Perhaps in support of the latter, mutant mice bearing defects in single‐positive thymocyte migration, such as CCR7‐ and PlexinD1‐deficient mice, have been reported to display ectopic medulla formation, including within subcapsular regions 29, 30. These data raise the possibility that TEC progenitor cells may additionally exist outside of the CMJ, or at the least that a fully formed CMJ is not essential for all mTEC precursor activity, and rather that it is the access of TEC precursors with latent mTEC potential to TNFSF‐ligand bearing thymocytes that is the essential regulatory factor in adult thymic medullary development and maintenance.

The study by Onder et al. 3 adds further insight into the developmental mechanisms regulating mTEC compartments, and raises additional questions that will continue to unravel the secrets of the thymic medulla. Further research will be required to define the overlap and potential hierarchy that may exist between multiple heterogeneous TEC precursor populations, including the contribution of bipotent versus unipotent TEC precursor activity in the adult thymus. In addition, whether a unipotent cTEC‐restricted precursor pool exists correspondingly exists in the adult thymus and maintains cortical compartments remains to be determined.

## Conflict of interest

The authors declare no financial or commercial conflict of interest.

**Abbreviations**
Aire: autoimmune regulator
cTEC: cortical thymic epithelial cell
CMJ: corticomedullary junction
mTEC: medullary thymic epithelial cell
MYHCA: myosin heavy chain alpha
NF‐kB: nuclear factor‐k B
NIK: NF‐kB‐inducing kinase
PDPN: podoplanin
RANK: receptor activator of nuclear factor‐k B
TNFSF: TNF superfamily
